# Paula Modersohn-Becker, the challenges of pregnancy and the weight of tradition

**DOI:** 10.1186/1747-5341-6-11

**Published:** 2011-06-06

**Authors:** Giorgina B Piccoli, Scott L Karakas

**Affiliations:** 1 SS Nephrology, Department of Clinical and Biological Sciences, University of Torino, TO, Italy; 2 Office of Curriculum and Instruction, Florida Gulf Coast University, Fort Myers, FL, USA

## Abstract

Paula Modersohn-Becker, widely considered to have been one of the most important independent Expressionist painters of the early twentieth century, was thirty-one years old when she gave birth to her first child. Following the then-common practice of putting women to bed rest for two-four weeks after delivery, she died of massive pulmonary embolism when she was first allowed to stand, eighteen days after giving birth. Paula had foreseen her death at a young age and was apprehensive about her pregnancy, yet she painted herself as pregnant in her best known self-portrait, thus underlining the importance of the pregnancy in her life. In the light of knowledge available at the time, the authors present a brief discussion of the life and death of Paula Modersohn-Becker as a reflection on the potential dangers of blindly following conventional wisdom in the medical profession.

## Introduction

"Wie schade" she said, and then she died. Thus on November 21, 1907, ended the brief life of Paula Modersohn-Becker at just thirty-one years of age. Widely considered to have been one of the most important independent Expressionist painters of the early twentieth century, Paula had given birth to her first child, Mathilde, on November 2, 1907. Her sudden death, due to massive pulmonary thromboembolism, occurred almost immediately after she was allowed to leave her bed for the first time following her delivery. Her biographers recount that she combed her hair, adorned it with red roses received as presents, and slowly walked to the living room, where her daughter was in her crib. Paula took Mathilde in her arms and said "Now it's almost as beautiful as Christmas", then suddenly fell to the floor. "What a pity" were her last words [1].

Paula's story demonstrates the deadly outcome of deep venous thrombosis, a complication of pregnancy that is relatively common when women are set to bed for a long time after delivery, as was customary at that time [2-4]. Now, some one hundred years later, the story of this very special young woman offers insights into the cultural reception of pregnancy, the changing definition of high-risk pregnancies, and the role of conventional wisdom in the medical profession.

### Paula's Life and Art

Paula Becker was born on February 8, 1876 in Dresden, Germany [5]. The third of seven children, Paula's mother came from an aristocratic family. Her Russian-born father served as an official with the German railway system, and the children grew up in a home stressing intellectualism and culture. Paula displayed artistic talent from a very early age, and her parents arranged for her to take painting and drawing lessons. Concerned that art would be a difficult career for a woman, they also encouraged her to complete a two-year teacher training course before agreeing to support her studies at the Society of Women Artists in Berlin, as well as the artists' colony at Worpswede. There she developed a close intellectual friendship with the poet Rainer Maria Rilke, who wrote his famous poem "Requiem for a Friend" in Paula's honor following her untimely death [6].

In 1901, Paula married fellow Worpswede painter Otto Modersohn. During extended visits to Paris, she was heavily influenced by the work of Cézanne and Gauguin. During her nine years as a working artist, Paula created more than four hundred paintings, and at least one thousand drawings and graphic works [7]. During her lifetime, her work was both ridiculed and praised, but was also largely overshadowed by that of her husband, Otto. In the years since her death, Paula's work has come to be regarded as of much greater importance to the history of art. In many ways, Paula was a forerunner of our age. In an era when young cultivated women were taught to play a little piano, cook creatively or paint a few watercolours, Paula stands out for her search for freedom and her uncommon spirit of adventure, together with her gift for painting.

**Figure 1:**
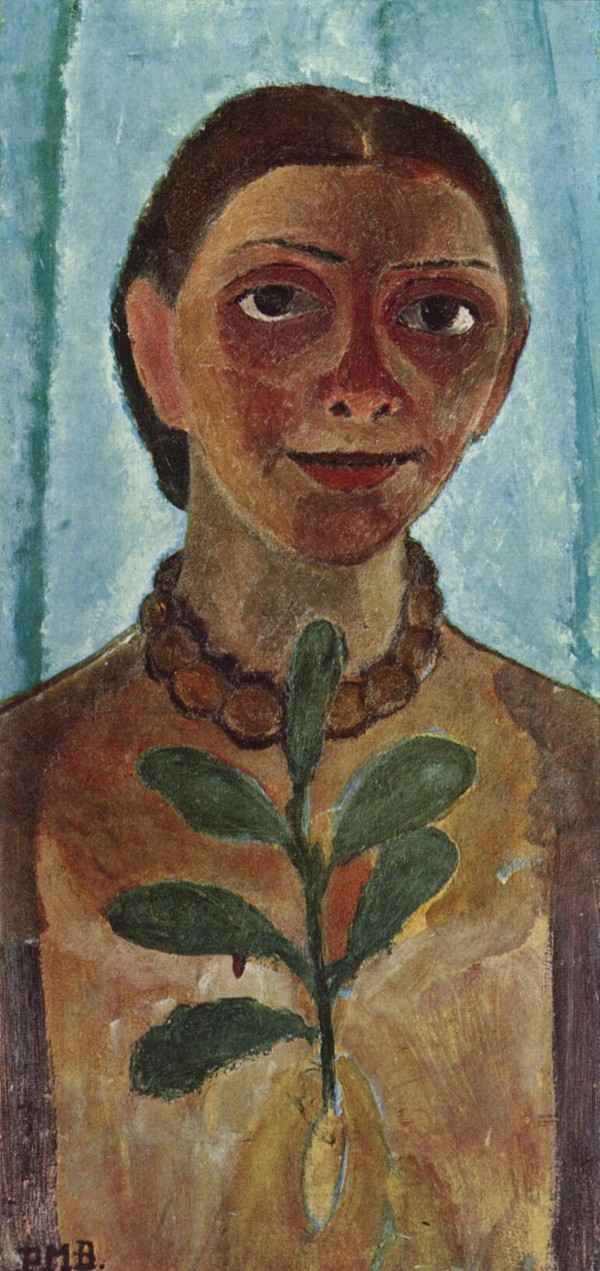
**Paula Modersohn Becker, *Die Malerin mit Kamelienzweig (Selbstporträt)*, 1907**. Museum Folkwang, Essen. The Yorck Project: *10.000 Meisterwerke der Malere: *http://commons.wikimedia.org/wiki/File:Paula_Modersohn-Becker_006.jpg. The work of art depicted in this image and the reproduction thereof are in the public domain worldwide. The reproduction is part of a collection of reproductions compiled by The Yorck Project. The compilation copyright is held by Zenodot Verlagsgesellschaft mbH and licensed under the GNU Free Documentation License http://www.gnu.org/licenses/fdl.html

**Figure 2:**
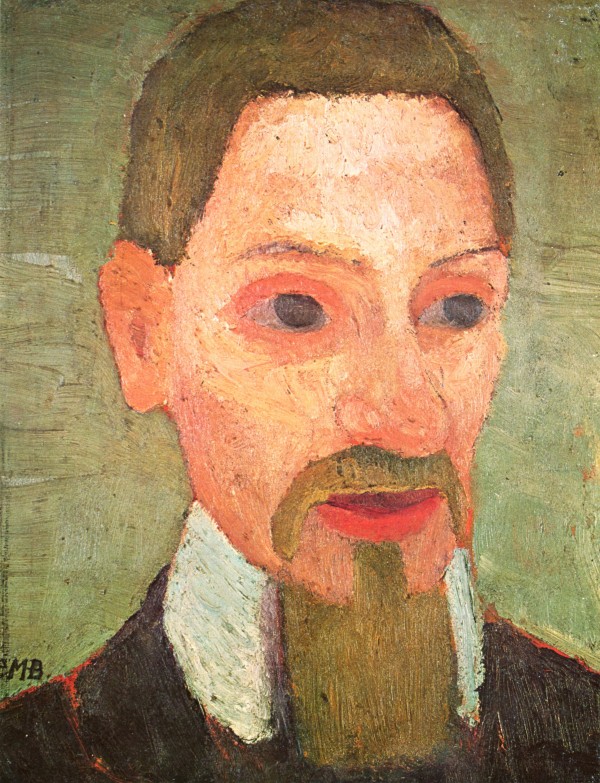
**Paula Modersohn-Becker, *Porträt des Rainer Maria Rilke*, 1906**. Sammlung Ludwig Roselius, Bremen. The Yorck *Project 10.000 Meisterwerke der Malerei: *http://commons.wikimedia.org/wiki/File:Paula_Modersohn-Becker_016.jpg. The work of art depicted in this image and the reproduction thereof are in the public domain worldwide. The reproduction is part of a collection of reproductions compiled by The Yorck Project. The compilation copyright is held by Zenodot Verlagsgesellschaft mbH and licensed under the GNU Free Documentation License http://www.gnu.org/licenses/fdl.html

According to her photographs and self-portraits, Paula Modersohn-Becker appears to have been a mixture of determination and shyness, as her bright look and slightly inclined head would suggest (figure 1). This is supported by Paula's journal entries and personal letters, which suggest a youthful, strong-willed, unconventional woman. Paula's letters and journals indicate that she was searching for an identity beyond the expectations of a traditional female role [8]. Paula considered Rainer Maria Rilke to be one of her best friends, a brother in the search for the meaning of art (figure 2). During her sojourn to Paris in 1906, Paula concluded one of her letters to Rilke with:

And now, I don't even know how I should sign my name. I'm not Modersohn, but I'm no longer Paula Becker anymore either. I am Me, and I hope to become Me more and more. That is surely the goal of all our struggles. *Letter to Rainer Maria Rilke, February 17, 1906 *[9].

A plaque at the foot of the stairs of the small museum in Bremen that houses many of Paula's paintings, states that this is the first museum in Europe dedicated to a female painter. That is, a male painter is a painter, while for a female painter the emphasis is on being a female, even in our times. After reading her memoirs, we suppose that she would say something like "it is not important being a female, it is important being a painter". However, in spite of, or because of, her deep comprehension of the limitations and rules of being a woman, and of her search for being "just" an artist, her life story is also a female story, whose steps are marriage, pregnancy and death.

### Paula's Pregnancy and Her Times

The private life of Paula Modersohn-Becker was spent in a difficult balance between a cultivated and rich German family, following the rules and restrictions of late nineteenth century European culture, and the strong desire for knowledge and independence. In late 1898 she wrote in her journal that:

I want to go further and further. I can hardly wait until I am a real artist.

And then I long so for life. I've only begun to get a little taste of it.

*Journal entry, December 1898 *[10].

Paula needed her family's approval and economic support to study art, which her father apparently viewed as little more than a hobby. She married one of her teachers, Otto Modersohn, who followed her, loved her, and nearly lost her. Almost two years before her death, Paula fled to Paris, trying to live alone there, but in the end returned to live with again with her husband. It is probably more than a coincidence that her return to Otto resulted in a pregnancy.

As in Paula's day, pregnancy still tends to define the ages of a woman's life. Interestingly the indication of high-risk pregnancy varies according to both biology and culture. At Paula's time, bearing a first child over age 30 was considered late and risky; the age of high-risk pregnancy has moved progressively from the early to the late thirties, and then to the present shift towards the forties [11].

Time for pregnancy became a leitmotif in Paula's painting, as probably also in her life. She also had a very strong sense, almost an obsession, of time and loss, and a strong premonition that her life was going to be short. In an oft-quoted sentence from her journal, she wrote:

As I was painting today, some thoughts came to me and I want to write them down for the people I love. I know that I shall not live very long. But I wonder, is that sad? Is a celebration more beautiful because it lasts longer? And my life is a celebration, a short, intense celebration.

*Journal entry, July 26, 1900 *[11].

The discrepancy between the best "biological" time for pregnancy and the best time for her personal achievements is quite clear from her letters. Marriage means children, while freedom means not having children: she wrote to Otto: "I cannot come back to you. Not yet.... I do not yet want to have a child by you. I must wait, if it comes again, or if something else comes out of it... [1]". Indeed, when Otto came to visit her in Paris and spent a few months in winter with her there, she ultimately returned home with him. The biographers describe her as quieter, but a bit sad, or somehow disheartened [2]. Maybe Paula was also pleased, coming back home, but none of the reports described her as happy and joyful. She was pregnant.

### Children and Pregnancy in Paula's Work

**Figure 3:**
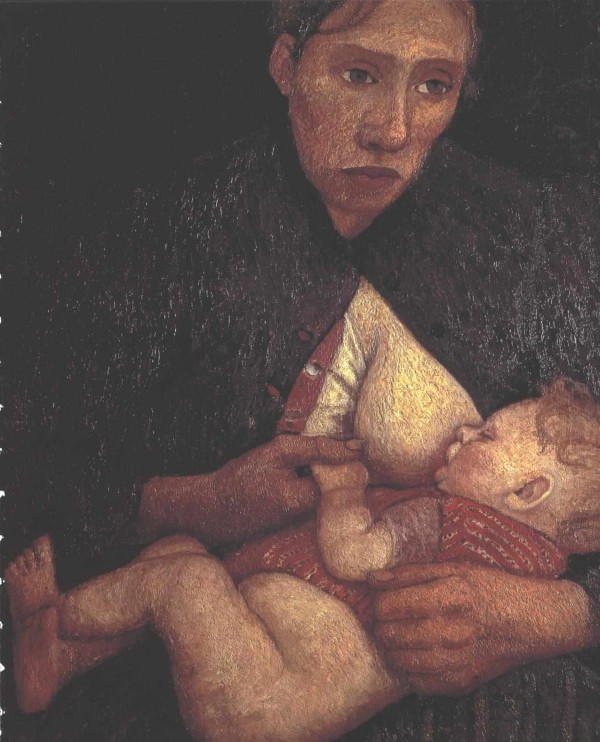
**Paula Modersohn-Becker, *Stillende Mutter*, 1903**. Niedersächsisches Landesmuseum, Hanover. Wikimedia: http://commons.wikimedia.org/wiki/File:Modersohn-Becker_-_Stillende_Mutter.jpg The work of art depicted in this image and the reproduction thereof are in the public domain worldwide. The reproduction is part of a collection of reproductions compiled by The Yorck Project. The compilation copyright is held by Zenodot Verlagsgesellschaft mbH and licensed under the GNU Free Documentation License http://www.gnu.org/licenses/fdl.html

Although heavily influenced by the work of contemporary Post Impressionists such as Les Nabis, Maillol, Rodin, van Gogh, Cézanne, and Gauguin, Paula Modersohn-Becker also demonstrated individualism and creativity in her art. She often portrayed nudes: women, children, girls, and sometimes boys. She also did numerous self portraits, sometimes dressed up and adorned with gold, pearl or amber necklaces, or often in the nude. Many of her paintings depict motherhood and the inexplicable mother-child bond (figure 3), and when Paula became pregnant, she rendered a number of self portraits in that condition. Although lacking the psychological intensity found in the self portraits of van Gogh, for example, Paula does appear to have used the form as a vehicle for self-exploration [12].

The structure of her paintings is grounded in the prevalent styles of her time. The colours and the often rough texture remind us of the Impressionists she knew well, and the background painted with separate colours as well as the impossible perspectives clearly show the lessons learnt from Gauguin and Cezanne, two contemporary masters Paula worshipped. However, unlike other female painters, such as Mary Cassatt, her mothers and children are full of defects, with red hands and noses, and sometimes too thin chins; in this, they partly acknowledge the lesson of German realism, but, in contrast to the latter, they are somehow embedded in melancholy, in a sort of ill-defined mist (figure 3). Paula seeks emotion, not beauty. She looks for the strains and strengths of reality, the sense of loss, the taste of age and disease. Many of the children she paints look sick, pale white or melancholic; when painted in summer, or naked, they display red hands and faces, from the open air, and white arms or bodies.

**Figure 4:**
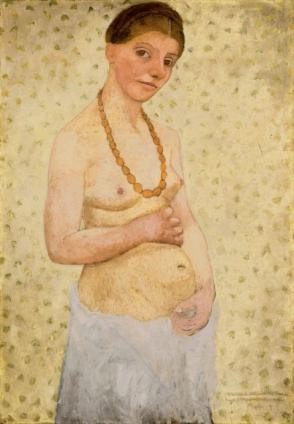
**Paula Modersoh-Becker, *Selbstbildnis am 6. Hochzeitstag*, 1906**. Paula Modersohn-Becker Museum, Bremen. Wikimedia:http://commons.wikimedia.org/wiki/File:Paula_Moderson-Becker_-_Selbstbildnis_am_6_Hochzeitstag_-_1906.jpeg The work of art depicted in this image and the reproduction thereof are in the public domain worldwide. The reproduction is part of a collection of reproductions compiled by The Yorck Project. The compilation copyright is held by Zenodot Verlagsgesellschaft mbH and licensed under the GNU Free Documentation License http://www.gnu.org/licenses/fdl.html

The delicate balance between beauty and truth, imagination and reality may be found in one of her most famous self-portraits, the symbol of the museum dedicated to her and possibly also the symbol of her complex relationship with pregnancy: her self-portrait painted on her 30^th ^birthday (figure 4). Here, Paula is half naked, with a white cloth around her hips and the long amber necklace she wears in other self-portraits. The background pattern is light and almost matches the light pale colour of her skin, except for her hands and face which are of a darker hue, similar to the way she depicted young farmers. As she was born on February 8, 1876 and as Mathilde was born on November 2, 1907, she was still not pregnant on the date of her thirtieth birthday. Yet, the painting definitely plays with the suggestion of four-five months pregnancy, the hands folded to protect the growing belly. And Paula measures time with pregnancy, painting a symbolic child marks a turning point for her.

### Wie schade. Could Her Death Have Been Avoided?

**Figure 5:**
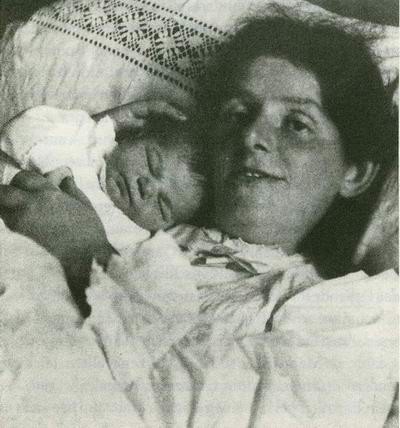
***Paula Modersohn-Becker with her daughter Mathilde*, November 1907**. Paula Modersohn-Becker Stiftung, Bremen. Wikipedia: http://commons.wikimedia.org/wiki/File:Gc-paula.jpg.

Paula was a healthy, albeit melancholic child, who grew up into a beautiful young lady. When she became pregnant, she had no "risk factor." She was fit and athletic (and there are some funny drawings by Otto depicting her morning gymnastics, her feet under the cupboard, her slightly round belly, her rather thin shoulders and nice breasts), was not overweight, and did not suffer from any chronic disease. The delivery was described as long and with some worries, as the doctor was unable to detect the child's heartbeat at one point and feared the child would be born dead. Yet Mathilde was born as a healthy child and the mother was soon well enough to receive visits from her mother and friends, including Rilke and his wife [13]. Nevertheless, she was kept in bed for eighteen days before being allowed to arise and move. All the photographs depict her lying in bed with her little daughter, happy, but also a bit tired (figure 5). Before getting out of bed, she combed her hair to signify that she was returning to life, to the life of a young woman who wanted to be pretty. She put the roses in her hair, as many times in the past she had painted children or young women with flowers in their hair [14].

A hundred years later, as physicians, we may wonder if her death was avoidable, and as art lovers we may wonder if she would have become a full-time mother, leaving aside the dream of painting, or, as her strong-minded letters and diaries suggest, if she would have made a substantial contribution to the history of art in the twentieth century. These questions are important to the understanding of both Paula's life and the dangers of conventional wisdom. At the time of Paula's death, the habit of putting women to complete bed rest was very well established, and difficult to change. While it may be difficult to date its origin, the first report by Otto Küstner, a German obstetrician, suggesting that four weeks of bed rest were probably too many for a healthy woman was considered almost a scandal in 1899 [4]. The practice at that time allowed sitting up in bed for no more than an hour in the first days; lying in bed for at least ten days was considered "a time honoured practice" in the early 1890s [2,3], despite the risks to the mother's health. Was Paula's physician a follower of Otto Küstner, she could probably have painted for a much longer time.

Likewise, the passage from the squatting position for delivery, still used in several aboriginal societies, to the so called gynaecological one, probably has complex sociological origins [15]. Moreover, the imposition of breast-feeding the baby at fixed times, devoid of physiological advantages, probably originates from the organizational needs of the first large factories, where women, in the absence of labour laws, needed to follow strict working schedules. And what is there to say about the baby face-up "revolution", which has completely changed the way of putting children to sleep in order to avoid suffocation and sudden infant death syndrome, after generations of nurses, nannies, mothers and grandmothers had put babies to sleep on their bellies? [16].

While probably in no field more than in obstetrics are myths and facts entangled, the history of medicine is full of such examples. While we praise the few courageous individuals, such as Dr. Küstner, who observed long-lasting habits, criticised them, understood their reasons and searched for adequate corrections, the story of Paula Modersohn-Becker may help us reflect on the meaning of art, the desire of posterity, the fear of death, the female role in our past and present society, and may give a lesson in humility, suggesting that we, as physicians, should reflect more on our habits and on the continuous changes of what previously appeared to be "well known".

## Competing interests

The authors declare that they have no competing interests.

## Authors' contributions

GB conceived and developed the paper topic, and wrote the first draft. SK contributed biographical and art historical material, and edited the manuscript. All authors read and approved the final manuscript.
